# *Candida* Administration Worsens Uremia-Induced Gut Leakage in Bilateral Nephrectomy Mice, an Impact of Gut Fungi and Organismal Molecules in Uremia

**DOI:** 10.1128/mSystems.01187-20

**Published:** 2021-01-12

**Authors:** Wimonrat Panpetch, Chitrasak Kullapanich, Cong Phi Dang, Peerapat Visitchanakun, Wilasinee Saisorn, Jutamas Wongphoom, Dhammika Leshan Wannigama, Arthid Thim-uam, Kanitha Patarakul, Naraporn Somboonna, Somying Tumwasorn, Asada Leelahavanichkul

**Affiliations:** a Department of Microbiology, Faculty of Medicine, Chulalongkorn University, Bangkok, Thailand; b Department of Microbiology, Faculty of Science, Chulalongkorn University, Bangkok, Thailand; c Microbiome Research Unit for Probiotics in Food and Cosmetics, Chulalongkorn University, Bangkok, Thailand; d Medical Microbiology, Interdisciplinary Program, Graduate School, Chulalongkorn University, Bangkok, Thailand; e Department of Pathology, King Chulalongkorn Memorial Hospital, Thai Red Cross Society, Chulalongkorn University, Bangkok, Thailand; f Translational Research in Inflammation and Immunology Research Unit (TRIRU), Department of Microbiology, Chulalongkorn University, Bangkok, Thailand; g Division of Biochemistry, School of Medical Sciences, University of Phayao, Phayao, Thailand; h School of Medicine, Faculty of Health and Medical Sciences, The University of Western Australia, Nedlands, Western Australia, Australia; i Antimicrobial Resistance and Stewardship Research Unit, Faculty of Medicine, Chulalongkorn University, Bangkok, Thailand; University of Hawaii at Manoa

**Keywords:** *Candida albicans*, bilateral nephrectomy, uremia, gut microbiota, gut leakage, probiotics

## Abstract

The impact of gut fungi and (1→3)-β-d-glucan (BG), a major fungal cell wall component, on uremia was explored by Candida albicans oral administration in bilateral nephrectomy (BiNx) mice because of the prominence of C. albicans in the human intestine but not in mice. As such, BiNx with *Candida* administration (BiNx-*Candida*) enhanced intestinal injury (colon cytokines and apoptosis), gut leakage (fluorescein isothiocyanate [FITC]-dextran assay, endotoxemia, serum BG, and bacteremia), systemic inflammation, and liver injury at 48 h postsurgery compared with non-*Candida* BiNx mice. Interestingly, uremia-induced enterocyte apoptosis was severe enough for gut translocation of viable bacteria, as indicated by culture positivity for bacteria in blood, mesenteric lymph nodes (MLNs), and other organs, which was more severe in BiNx-*Candida* than in non-*Candida* BiNx mice. *Candida* induced alterations in the gut microbiota of BiNx mice as indicated by (i) the higher fungal burdens in the feces of BiNx-*Candida* mice than in sham-*Candida* mice by culture methods and (ii) increased *Bacteroides* with decreased *Firmicutes* and reduced bacterial diversity in the feces of BiNx-*Candida* mice compared with non-*Candida* BiNx mice by fecal microbiome analysis. In addition, lipopolysaccharide plus BG (LPS+BG), compared with each molecule alone, induced high supernatant cytokine levels, which were enhanced by uremic mouse serum in both hepatocytes (HepG2 cells) and macrophages (RAW264.7 cells). Moreover, LPS+BG, but not each molecule alone, reduced the glycolysis capacity and mitochondrial function in HepG2 cells as determined by extracellular flux analysis. Additionally, a probiotic, Lactobacillus rhamnosus L34 (L34), attenuated disease severity only in BiNx-*Candida* mice but not in non-*Candida* BiNx mice, as indicated by liver injury and serum cytokines through the attenuation of gut leakage, the fecal abundance of fungi, and fecal bacterial diversity but not fecal Gram-negative bacteria. In conclusion, *Candida* enhanced BiNx severity through the worsening of gut leakage and microbiota alterations that resulted in bacteremia, endotoxemia, and glucanemia.

**IMPORTANCE** The impact of fungi in the intestine on acute uremia was demonstrated by the oral administration of Candida albicans in mice with the removal of both kidneys. Because fungi in the mouse intestine are less abundant than in humans, a *Candida*-administered mouse model has more resemblance to patient conditions. Accordingly, acute uremia, without *Candida*, induced intestinal mucosal injury, which resulted in the translocation of endotoxin, a major molecule of gut bacteria, from the intestine into blood circulation. In acute uremia with *Candida*, intestinal injury was more severe due to fungi and the alteration in intestinal bacteria (increased *Bacteroides* with decreased *Firmicutes*), leading to the gut translocation of both endotoxin from gut bacteria and (1→3)-β-d-glucan from *Candida*, which synergistically enhanced systemic inflammation in acute uremia. Both pathogen-associated molecules were delivered to the liver and induced hepatocyte inflammatory responses with a reduced energy production capacity, resulting in acute uremia-induced liver injury. In addition, Lactobacillus rhamnosus attenuated intestinal injury through reduced gut *Candida* and improved intestinal bacterial conditions.

## Abstract (precis)

The impact of fungi in the intestine on acute uremia was demonstrated by the oral administration of Candida albicans in mice with the removal of both kidneys. Because fungi in the mouse intestine are less abundant than in humans, a *Candida*-administered mouse model has more resemblance to patient conditions.

## INTRODUCTION

Acute kidney injury, a major health care problem worldwide, is one of the main causes of death in intensive care units (1), at least in part due to uremia-enhanced inflammatory responses (2). In addition, uremia alters gut organisms, which is associated with gut permeability defects (gut leakage), leading to spontaneous endotoxemia from gut translocation (3, 4). Because a single layer of the gut epithelium is a permeability barrier with a 32-m^2^ surface area between the host and gut organisms (5), a gut permeability defect leads to the translocation of several organismal molecules from the gut into blood circulation (6, 7). Indeed, it was reported that the attenuation of gut leakage improved uremic complications in patients with chronic kidney disease (8). Nevertheless, not only are Gram-negative bacteria a source of endotoxin (lipopolysaccharide [LPS]) in the gut, but fungi, especially Candida albicans, are also predominant gut organisms in humans (9). Accordingly, the influence of gut fungi in several patient conditions has been mentioned, for example, the “*Candida* colonization index” in bacterial sepsis (10) and increased *Candida* colonization in the gut of patients with alcoholic liver cirrhosis (11–13). In addition, (1→3)-β-d-glucan (BG), a major *Candida* cell wall component, in serum from gut translocation enhances systemic inflammation through the additive effect on endotoxemia, as demonstrated in several models (14, 15), at least in part by the synergy of Toll-like receptor 4 (TLR-4) and Dectin-1, receptors of LPS and BG, respectively (16).

However, the influence of intestinal C. albicans in mouse models is not properly considered, as there is a lesser abundance of C. albicans in the mouse gut than in the human intestine (17). Indeed, the abundance of *Candida* spp. in mouse feces is not high enough to be detectable by stool culture (17), which is different from cultures of human feces (18). Although gut fungi do not seem to cause illness directly, they alter the gut microbiota and provide BG in the gut, contributing to the enhancement of systemic inflammation after gut leakage (19). Unfortunately, there are very few studies on the influence of gut fungi on uremic conditions, and analysis of a uremic model with C. albicans presentation has never been explored. Hence, we performed an acute uremia model in mice with preconditioning C. albicans administration before bilateral nephrectomy (BiNx) surgery (BiNx-*Candida* mice) to examine the impact of C. albicans on uremia. Interestingly, gut leakage could be directly inducible by uremia (20), which is possibly worsened by fungal presentation in the gut (6).

Probiotic use is a strategy to strengthen gut epithelial permeability/selectivity, possibly through microbiota alterations in the gut and the production of protective factors (21–23) that have been used against uremia (8). *Lactobacillus* species is one of the most common probiotics, with several antagonistic mechanisms against *Candida* (24), and Lactobacillus rhamnosus strain L34 (L34) demonstrates anti-inflammatory effects on both intestinal epithelial cells (25) and several murine models (15, 26, 27). Hence, L34 was also tested in our mouse model. We hypothesized that *Candida* administration would worsen inflammation in BiNx mice through alterations in the gut microbiota and enhanced gut leakage, which could possibly be attenuated by L34 administration. The influence of gut fungi and probiotics on acute uremia might lead to the utilization of gut fungi (or serum BG) in uremia, which supports using L34 as a new candidate treatment.

## RESULTS

### *Candida* administration induced liver injury, gut leakage, systemic inflammation, and microbiota alterations in feces of bilateral nephrectomy mice.

**FIG 1:**
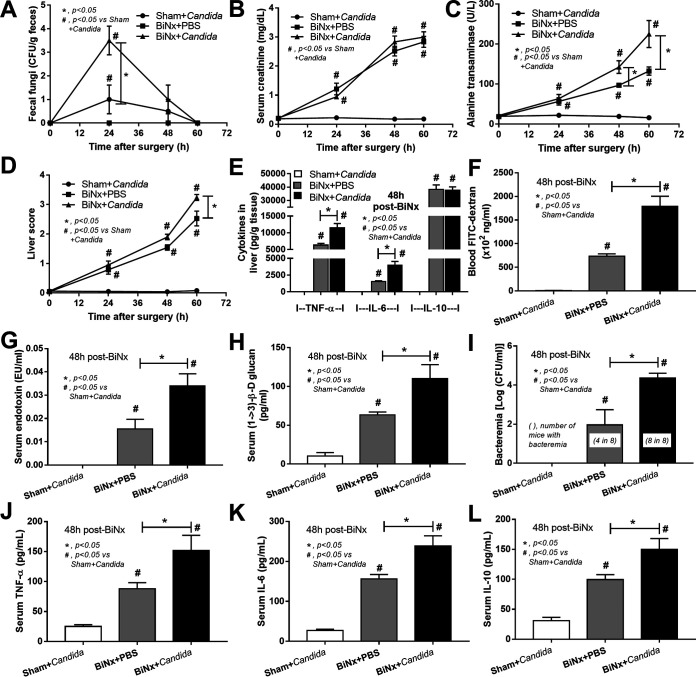
(A to D) Characteristics of mice evaluated by the time points of fecal fungi (A), serum creatinine (B), alanine transaminase (C), and liver histology score (D) in sham treatment with *Candida* administration (Sham+*Candida*) (*n* = 8/time point) or bilateral nephrectomy with PBS (BiNx+PBS) or with *Candida* administration (BiNx+*Candida*) (*n* = 7 to 8/time point at 0 to 48 h and *n* = 4/group at 60 h due to moribund conditions). (E to L) Liver cytokines (E); gut leakage as determined by FITC-dextran, endotoxemia, serum (1→3)-β-d-glucan, and bacteremia (F to I); and serum cytokines (J to L) (*n* = 7 to 8/group). Sham without *Candida* is not demonstrated due to the nondifference from Sham+*Candida*.

**FIG 2:**
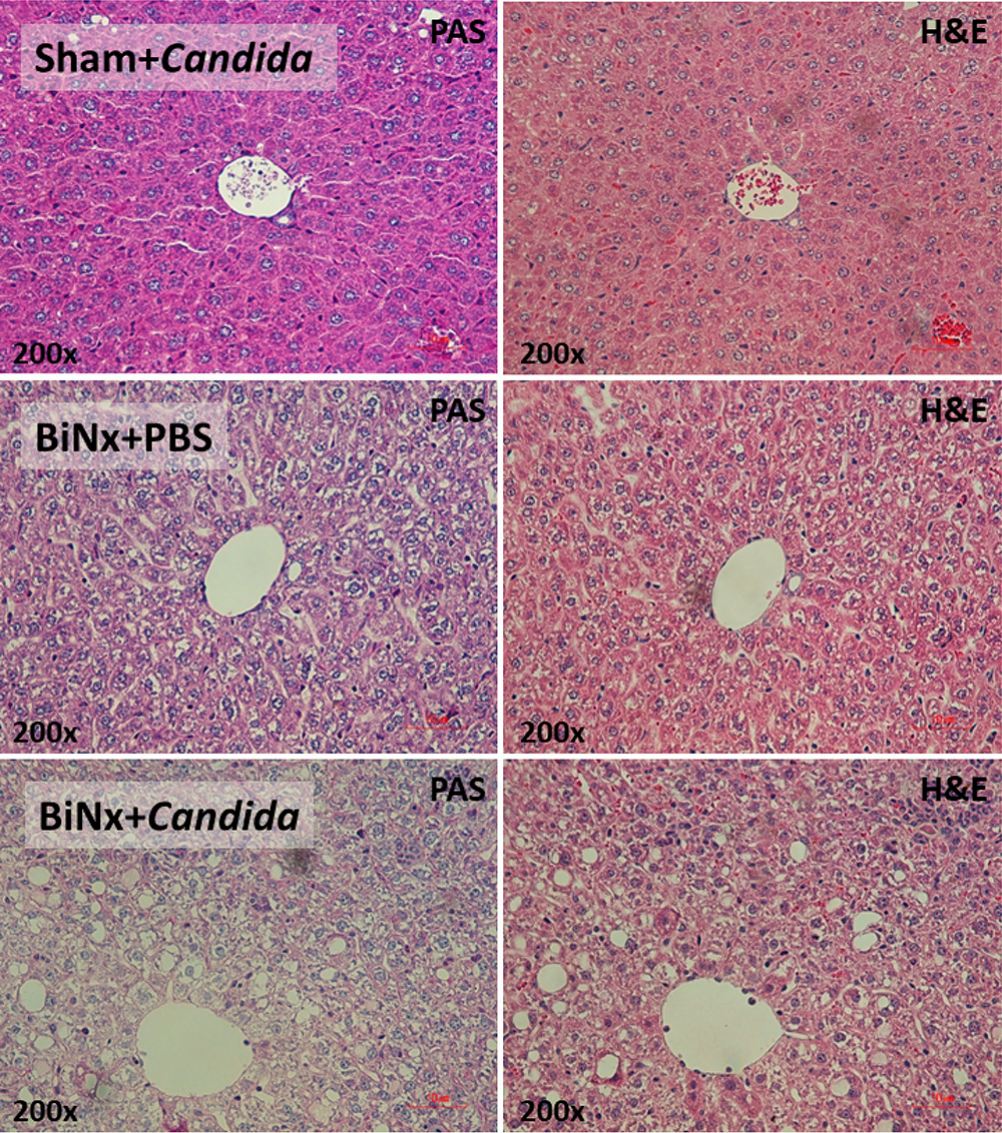
Representative pictures of liver histology of mice with sham or bilateral nephrectomy with PBS (BiNx+PBS) or with *Candida* administration (BiNx+*Candida*) at 60 h postoperation. Sham without *Candida* is not demonstrated due to the nondifference from Sham+*Candida*.

C. albicans administration transiently induced fecal fungi for ∼48 h in both sham and BiNx mice, with the predominance in BiNx mice (Fig. 1A) supporting that uremia induced the alterations in the gut microbiota (28). While *Candida* administration did not increase serum creatinine, liver enzymes and liver injury scores were enhanced as early as 48 h and 60 h after BiNx, respectively, in comparison with BiNx alone (Fig. 1B to D and Fig. 2). In addition, the liver tissue cytokines tumor necrosis factor alpha (TNF-α) and interleukin-6 (IL-6), but not IL-10, were also higher 48 h after BiNx in BiNx-*Candida* mice (Fig. 1E). Moreover, *Candida* also enhanced gastrointestinal permeability defects (gut leakage) 48 h after BiNx as determined by a fluorescein isothiocyanate (FITC)-dextran assay and the spontaneous elevation of LPS and BG in serum and bacterial burdens in blood (Fig. 1F to I). Of note, bacteremia presented in one-half and all mice from the BiNx-alone and BiNx-*Candida* mouse groups, respectively (Fig. 1I). The presentations of LPS, BG, and bacteremia in blood and uremia-related systemic inflammation (serum cytokines) of BiNx-*Candida* mice were greater than those with BiNx alone (Fig. 1J to L). Interestingly, all of these parameters between sham with *Candida* and sham without *Candida* were not different (data not shown), implying effective mucosal immunity against gut fungi in healthy mice.

**FIG 3:**
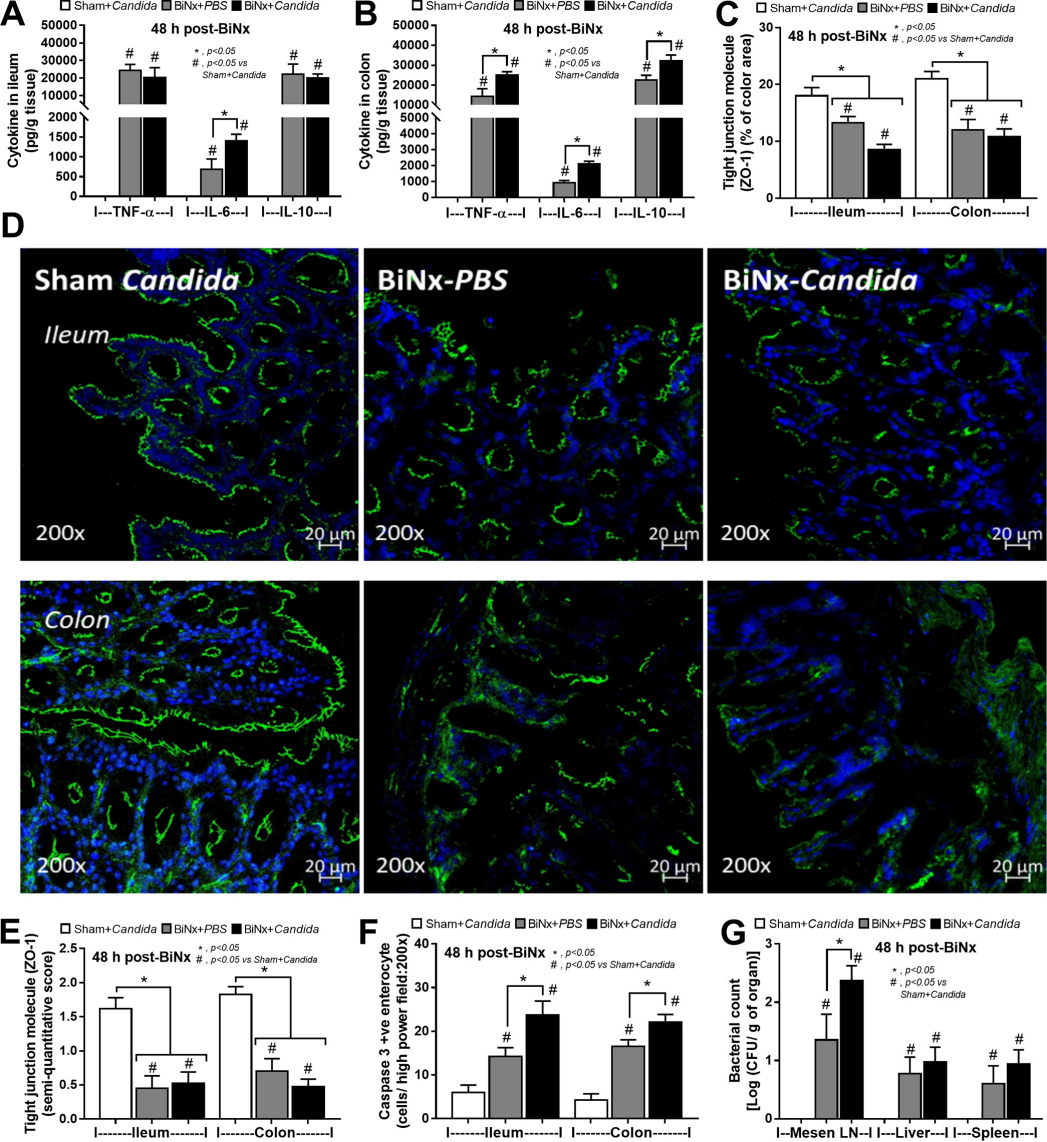
(A to F) Intestinal injury in the ileum and colon determined by intestinal cytokines (TNF-α, IL-6, and IL-10) (A and B), the tight junction molecule (zona occludens-1 [ZO-1]) presented by area of immunofluorescence staining with the representative figures (C and D) and immunohistochemistry scores (E), and enterocyte apoptosis by active caspase-3 immunohistochemistry staining (F) from mice with *Candida* administered sham (Sham+*Candida*) or bilateral nephrectomy with PBS (BiNx+PBS) or with *Candida* administration (BiNx+*Candida*) (*n* = 7 to 8/group). (G) Bacterial burdens in several organs of these mice (*n* = 6/group). Mesen LN, mesenteric lymph node. Sham without *Candida* is not demonstrated due to the nondifference from Sham+*Candida*.

**FIG 4:**
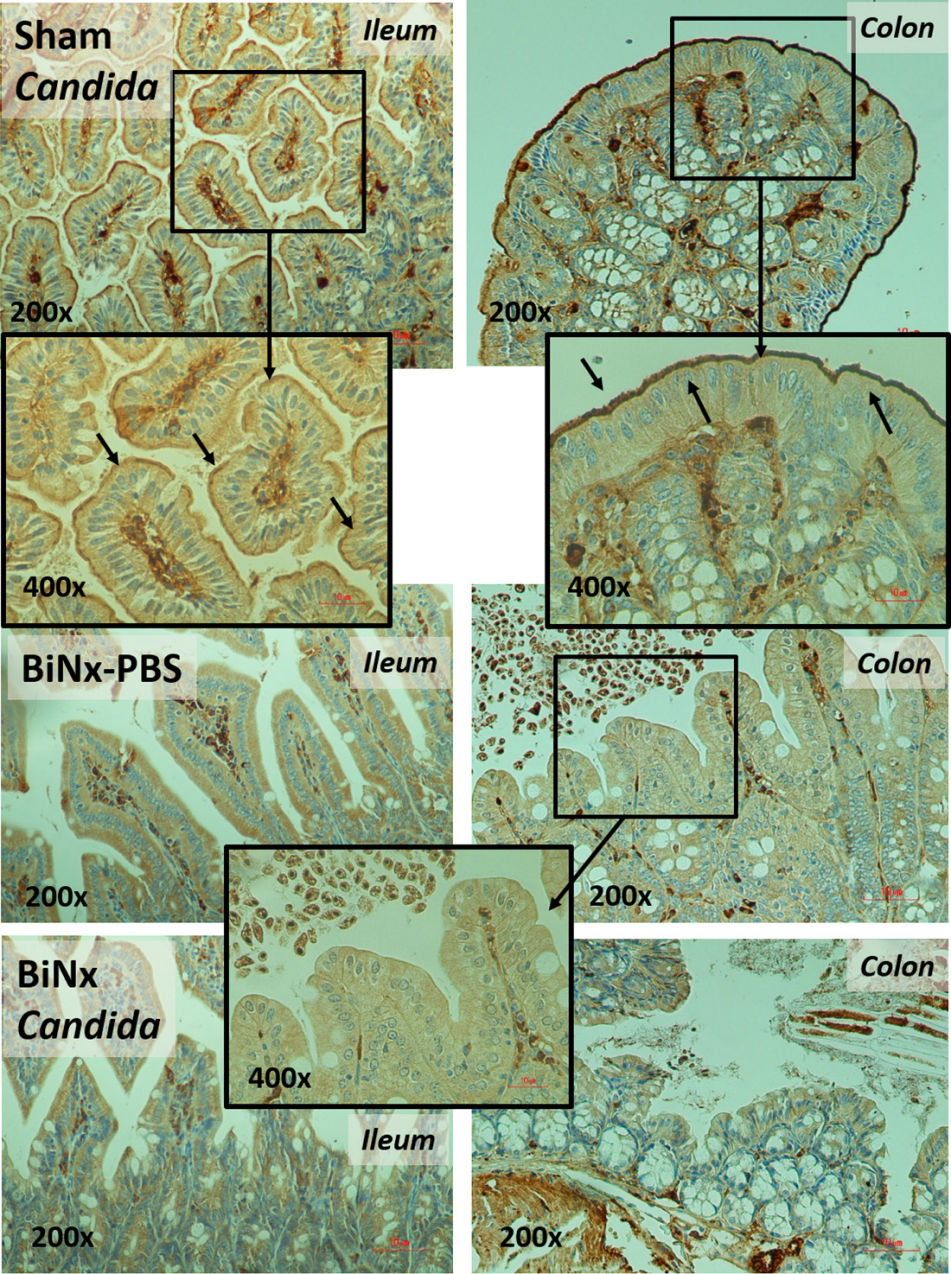
Representative immunohistochemistry pictures for the detection of zona occludens 1 (ZO-1), a tight junction molecule, in the ileum and colon from mice with *Candida* administered sham (Sham+*Candida*) or bilateral nephrectomy with PBS (BiNx+PBS) or with *Candida* administration (BiNx+*Candida*). Sham without *Candida* is not demonstrated due to the nondifference from Sham+*Candida*. The image at a ×400 magnification of the ileum and colon of sham-*Candida* mice demonstrates positive ZO-1 staining at the enterocyte villi (arrows), and the image at a ×400 magnification of the colon of BiNx mice indicates nondetectable staining.

**FIG 5:**
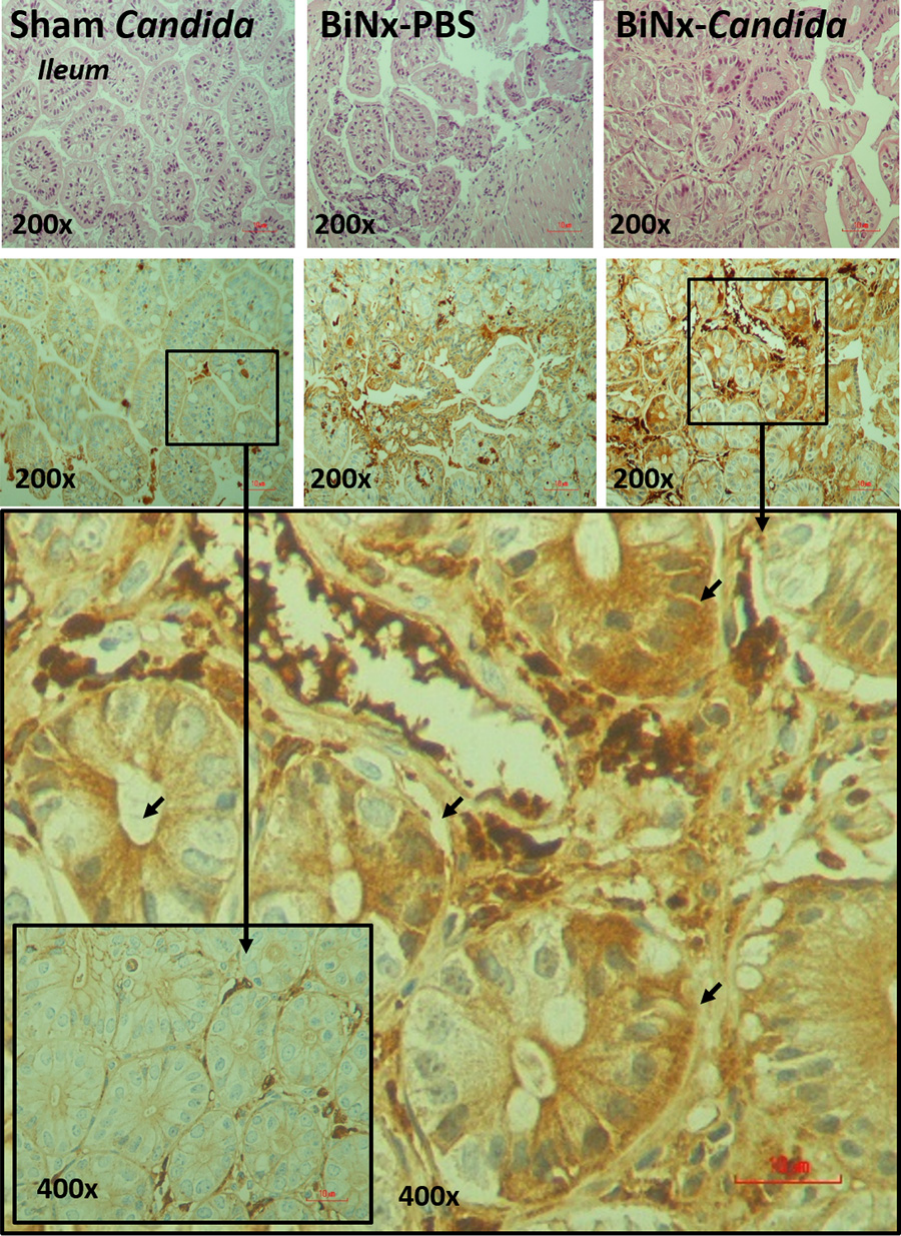
Representative pictures of hematoxylin and eosin (H&E) staining (top) and immunohistochemistry (bottom) for the detection of intestinal apoptosis from the ileum of mice with *Candida* administered sham (Sham+*Candida*) or bilateral nephrectomy with PBS (BiNx+PBS) or with *Candida* administration (BiNx+*Candida*). Sham without *Candida* is not demonstrated due to the nondifference from Sham+*Candida*. The image at a ×400 magnification of the ileum of BiNx-*Candida* and sham-*Candida* mice indicates positive (arrows) and negative staining for active caspase-3, respectively.

**FIG 6:**
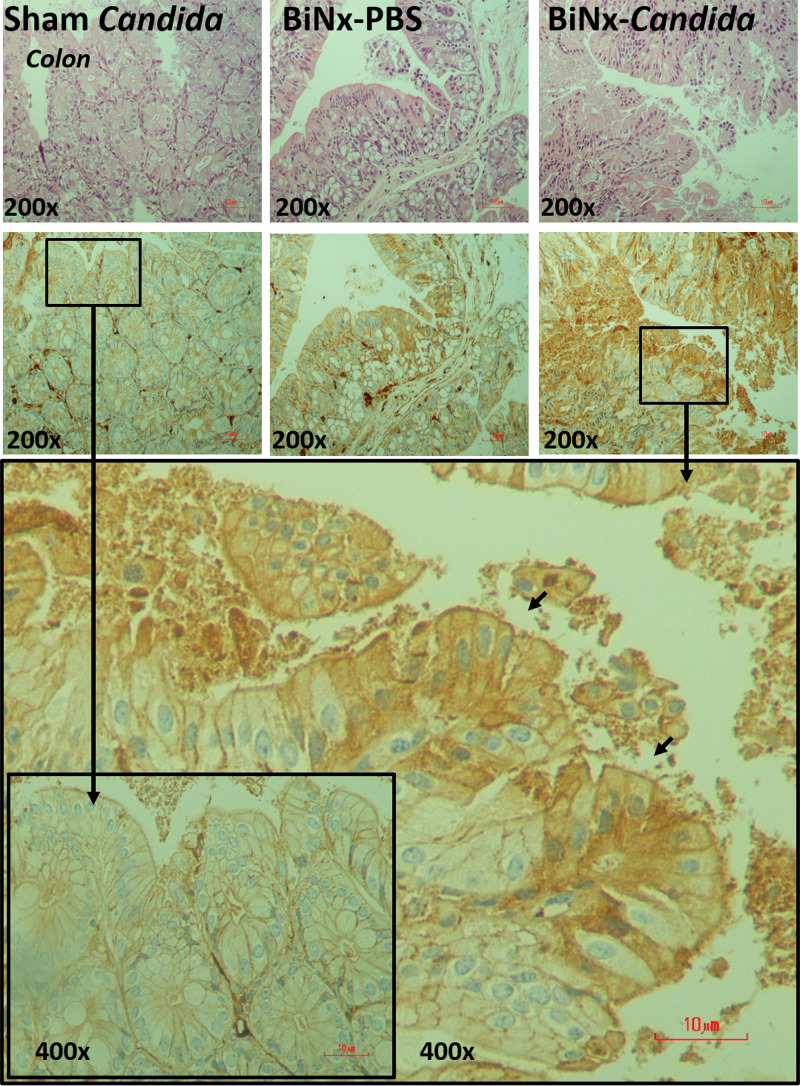
Representative pictures of hematoxylin and eosin (H&E) staining (top) and immunohistochemistry (bottom) for the detection of intestinal apoptosis from the colon of mice with *Candida* administered sham (Sham+*Candida*) or bilateral nephrectomy with PBS (BiNx+PBS) or with *Candida* administration (BiNx+*Candida*). Sham without *Candida* is not demonstrated due to the nondifference from Sham+*Candida*. The image at a ×400 magnification of the ileum of BiNx-*Candida* and sham-*Candida* mice indicates positive (arrows) and negative staining for active caspase-3, respectively.

In addition, local inflammation of the small bowel (ileum) and large bowel (colon) was evaluated by inflammatory cytokines from intestinal tissue. The levels of intestinal cytokines in BiNx mice, regardless of *Candida* administration, were higher than those in sham-*Candida* mice (Fig. 3A and B). The levels of only IL-6 in the ileum and all cytokines in the colon of BiNx-*Candida* mice were higher than those in mice with BiNx alone (Fig. 3A and B). In parallel, the tight junction molecules were decreased in BiNx mice regardless of *Candida* administration, without a difference between BiNx-*Candida* and BiNx alone (Fig. 3C to E and Fig. 4). However, BiNx induced enterocyte apoptosis, which was more prominent in BiNx-*Candida* than in BiNx-alone mice, as determined by the cleavage of caspase-3 (Fig. 3F and Fig. 5 and 6). Interestingly, there was no abnormality detected by intestinal hematoxylin and eosin (H&E) staining (Fig. 5 and 6, top) despite detectable apoptosis by caspase-3 immunohistochemistry staining (Fig. 5 and 6, bottom). The apoptosis in intestinal cells was possibly responsible for the gut translocation of viable bacteria, as presented by bacterial burdens in blood (Fig. 1I) and in several organs, including mesenteric lymph nodes (MLNs) (Fig. 3G). Of note, bacterial presentation in mesenteric lymph nodes supported viable bacterial translocation from the gut into blood circulation during gut leakage (6). However, the viable fungi were nonculturable from all of these organs (data not shown), possibly because fungi are >10-fold larger than bacteria (29), supporting data from previous studies (15, 30).

**FIG 7:**
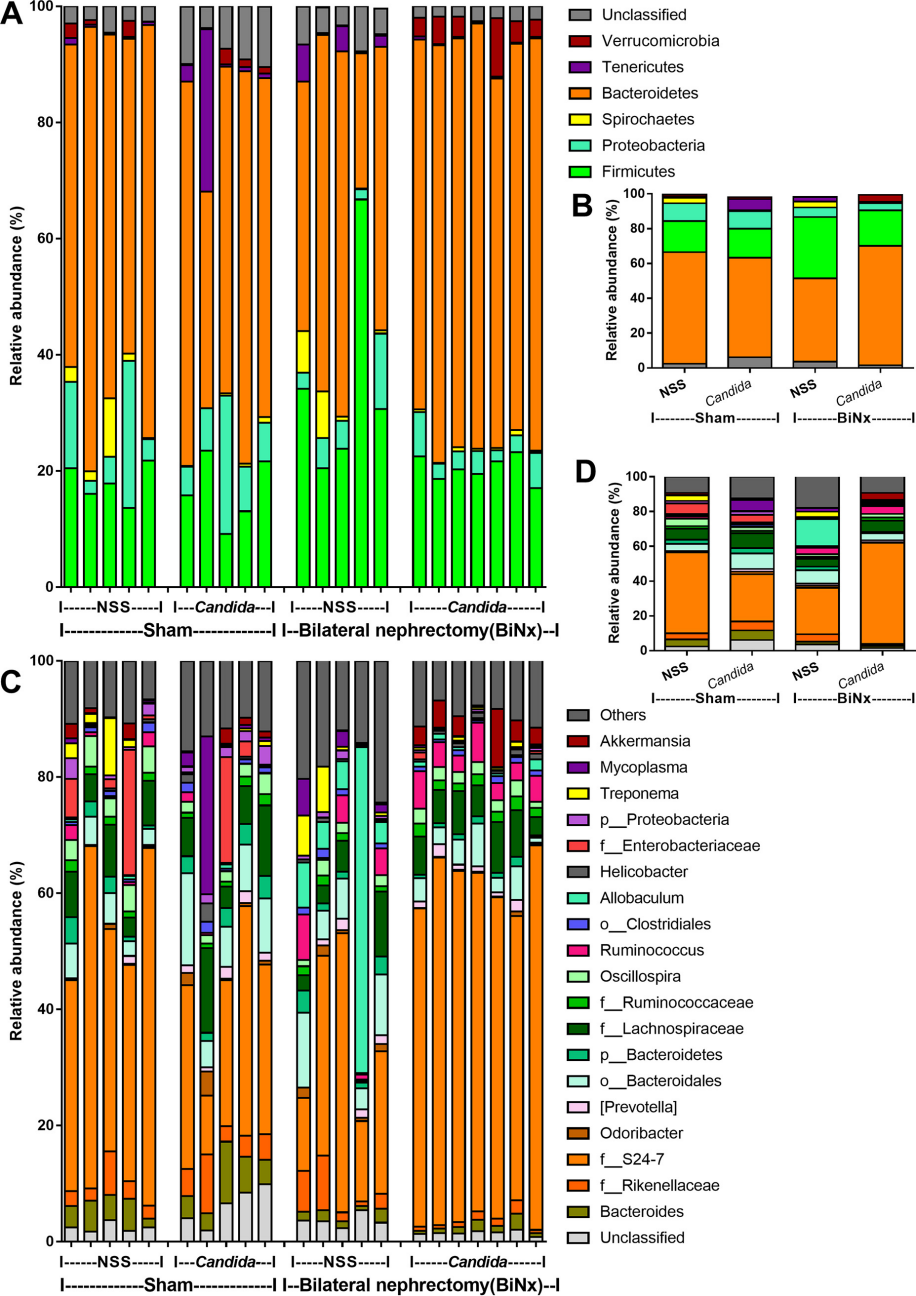
Fecal microbiota analysis of mice with sham or bilateral nephrectomy (BiNx) with normal saline (NSS) or *Candida* administration at 48 h postoperation by the relative abundance of bacteria at the phylum level, for all samples and by average (A and B), and at the genus level, for all samples and by average (C and D).

**FIG 8:**
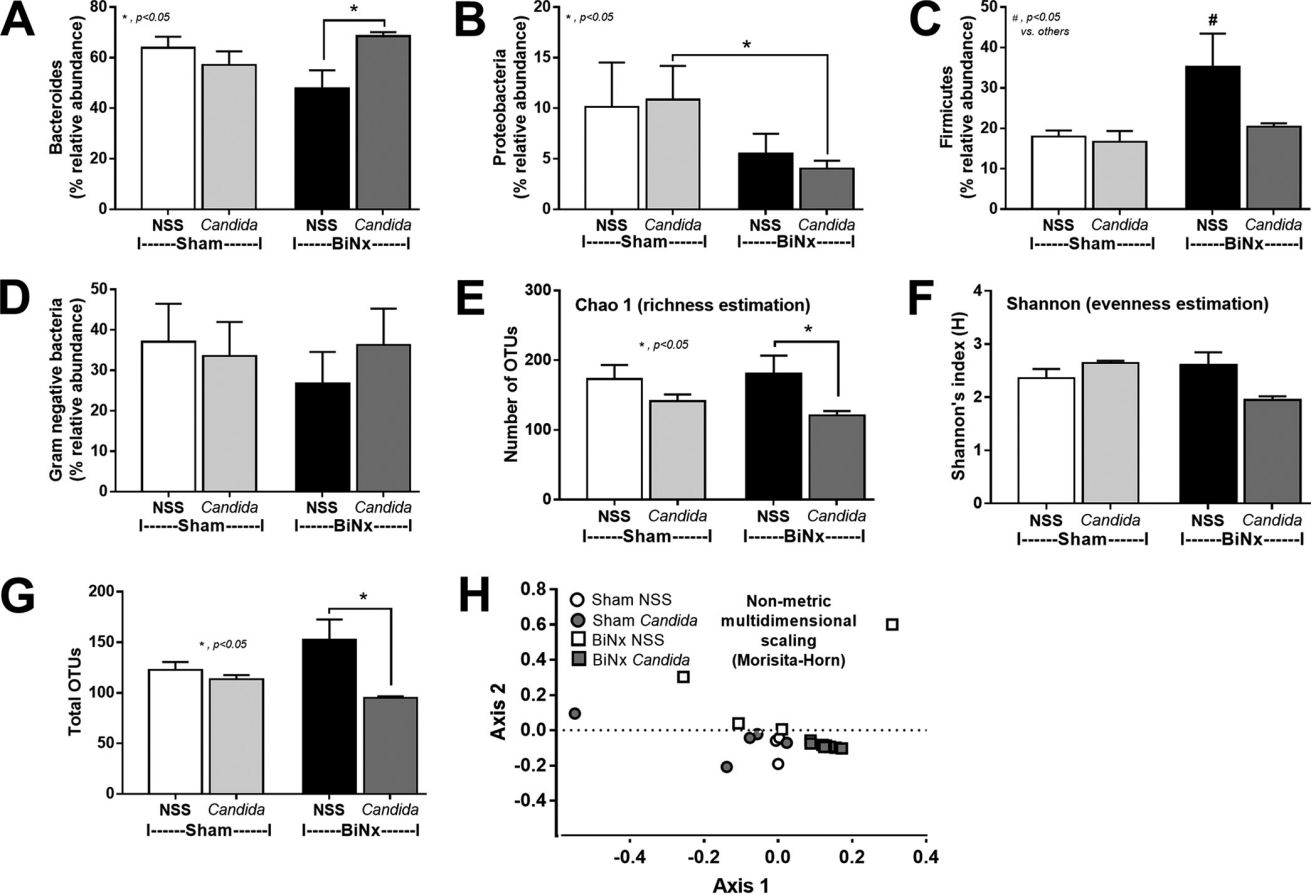
Fecal microbiota analysis of mice with sham or bilateral nephrectomy (BiNx) with normal saline (NSS) or *Candida* administration at 48 h postoperation presented as graphs of the relative abundances of bacteria at the phylum level, including *Bacteroides*, *Proteobacteria*, and *Firmicutes* (A to C); the abundances of total Gram-negative bacteria in feces as determined from bacterial phyla (D); the alpha diversity presented by Chao 1, Shannon index, and total operational taxonomic units (OTUs) (E to G); and the beta diversity (nonmetric multidimensional scaling based on Morisita-Horn dissimilarity indices) (H).

Because (i) alterations in the gut microbiota induce gut leakage (31, 32); (ii) gut *Candida* directly and indirectly damages the gut mucosa through fungal invasion and microbiota alterations, respectively (33); and (iii) Gram-negative bacteria in the gut are an endogenous source of endotoxins (34), the alterations in the gut microbiota of BiNx mice might be associated with the severity of gut leakage and endotoxemia. As such, fecal microbiome analysis indicated that *Candida* in sham mice did not significantly alter the fecal microbiome compared with non-*Candida* sham mice (Fig. 7 and 8). Meanwhile, BiNx reduced *Proteobacteria* (Gram-negative pathogenic aerobes) and increased *Firmicutes* (Gram-positive anaerobes) in feces (Fig. 7 and Fig. 8B and C) without alterations in other parameters (Fig. 8). On the other hand, *Candida* in BiNx, in comparison with BiNx alone, increased *Bacteroides* species (Gram-negative anaerobes), decreased *Firmicutes* (Fig. 7 and Fig. 8A and C), and reduced the total amount of fecal bacteria (Chao 1 richness estimation and total operational taxonomic units [OTUs]) (Fig. 8E and G), with a tendency toward a reduced variety of bacterial species (Shannon evenness estimation) (Fig. 8F). In parallel, there was a close proximity of taxonomic abundance profiles by β-diversity analysis (similar types of bacteria) only among feces of BiNx-*Candida* mice (Fig. 8H). Hence, *Candida* altered the gut microbiota in BiNx mice through (i) increased *Bacteroides* species, pathogenic Gram-negative anaerobes under some conditions (35, 36), and (ii) a reduced variety of bacterial species (Fig. 8).

### Additive effect between endotoxin and (1→3)-β-d-glucan toward hepatocytes and macrophages.

**FIG 9:**
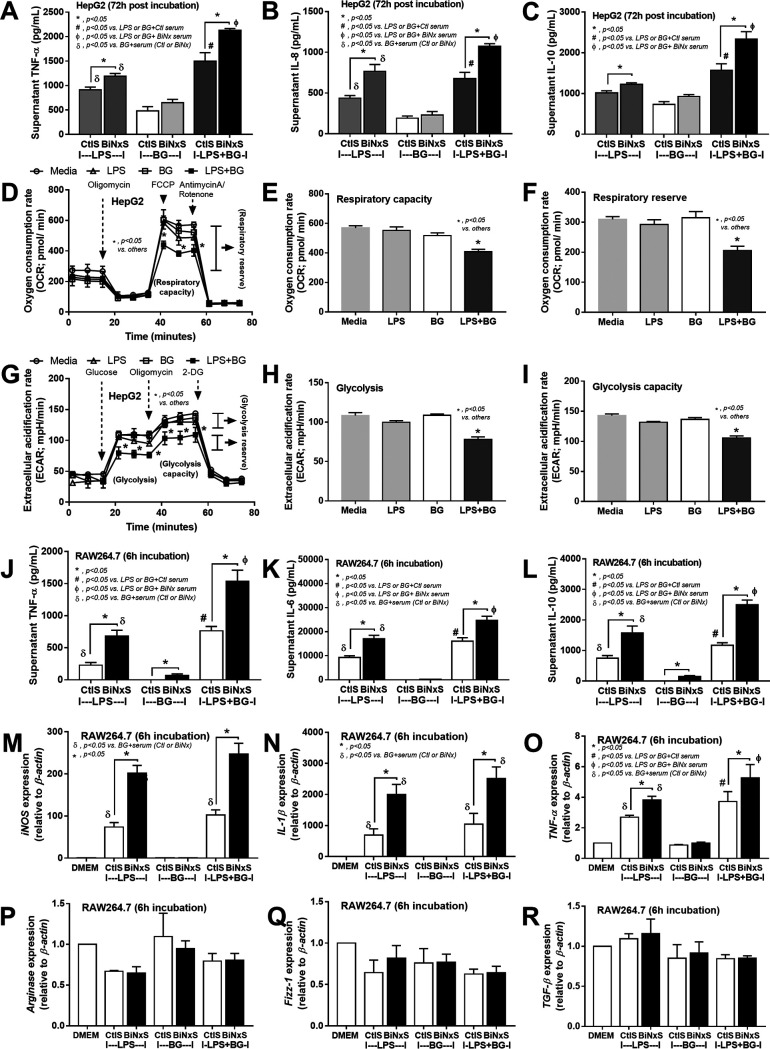
(A to C) Characteristics of HepG2 cells (hepatocyte cell line) after 72 h of incubation of endotoxin (LPS), (1→3)-β-d-glucan (BG), or LPS plus BG (LPS+BG) with mouse serum from the sham control group (control serum [CtlS]) or from the bilateral nephrectomy (BiNx) group (BiNx serum [BiNxS]) evaluated by supernatant cytokines. (D to I) Extracellular flux analysis patterns of HepG2 cells 48 h after activation with LPS, BG, and LPS+BG determined by the oxygen consumption rate from a mitochondrial stress test, respiratory capacity, and respiratory reserve (D to F) and by the extracellular acidification rate from a glycolysis stress test, glycolysis, and glycolysis capacity (G to I). 2-DG, 2-deoxy-d-glucose; FCCP, carbonyl cyanide-4-(trifluoromethoxy)phenylhydrazone. (J to R) Characteristics of RAW264.7 cells (macrophage cell line) after 6 h of incubation of LPS, BG, and LPS+BG with control mouse serum or BiNx serum evaluated by supernatant cytokines (J to L) and the expression of proinflammatory genes (*iNOS*, *IL-1*β, and *TNF-*α) (M to O) and anti-inflammatory genes (*Arginase-1*, *FIZZ-1*, and *TGF-*β) (P to R). (Independent triplicate experiments were performed.)

Elevated endotoxin (LPS) and (1→3)-β-d-glucan (BG) in BiNx mouse serum were amplified by *Candida* (Fig. 1G and H), possibly due to gut leakage and alterations in the gut microbiota (Fig. 1F and Fig. 7 and 8). Because the main translocation routes from the gut into blood circulation are via the portal vein (to the liver) and lymphatic system (to mesenteric lymph nodes), hepatocytes and macrophages are activated during gut translocation. As such, 24 h of incubation of BiNx mouse serum with LPS and LPS plus BG (LPS+BG) but not BG alone enhanced cytokine production in hepatocytes in comparison with incubation with control mouse serum (Fig. 9A to C). Although activation by BG alone showed a lesser impact on hepatocyte cytokine production, BG enhanced hepatocyte responses against LPS stimulation (Fig. 9A to C). Moreover, LPS+BG but not activation by each molecule alone interfered with mitochondria and glycolysis as LPS+BG-activated hepatocytes demonstrated the lowest respiratory capacity, respiratory reserve, glycolysis, and glycolysis reserve (Fig. 9D to I). In parallel, serum BiNx also enhanced macrophage inflammatory responses of LPS and LPS+BG as determined by supernatant cytokines and the gene expression of M1 macrophage polarization (*iNOS* [inducible nitric oxide synthase], *IL-1*β, and *TNF-*α), but not anti-inflammatory genes (*Arginase-1*, *FIZZ-1*, and *TGF*-β [transforming growth factor β]), in comparison with activation by control mouse serum (Fig. 9J to R). Of note, BG activation alone induced only low levels of inflammation (Fig. 9J to R). These data support the systemic inflammatory effects of LPS and BG from gut leakage and uremic serum against hepatocytes and macrophages.

### Probiotic treatment attenuates the severity of bilateral nephrectomy in mice with *Candida* administration.

**FIG 10:**
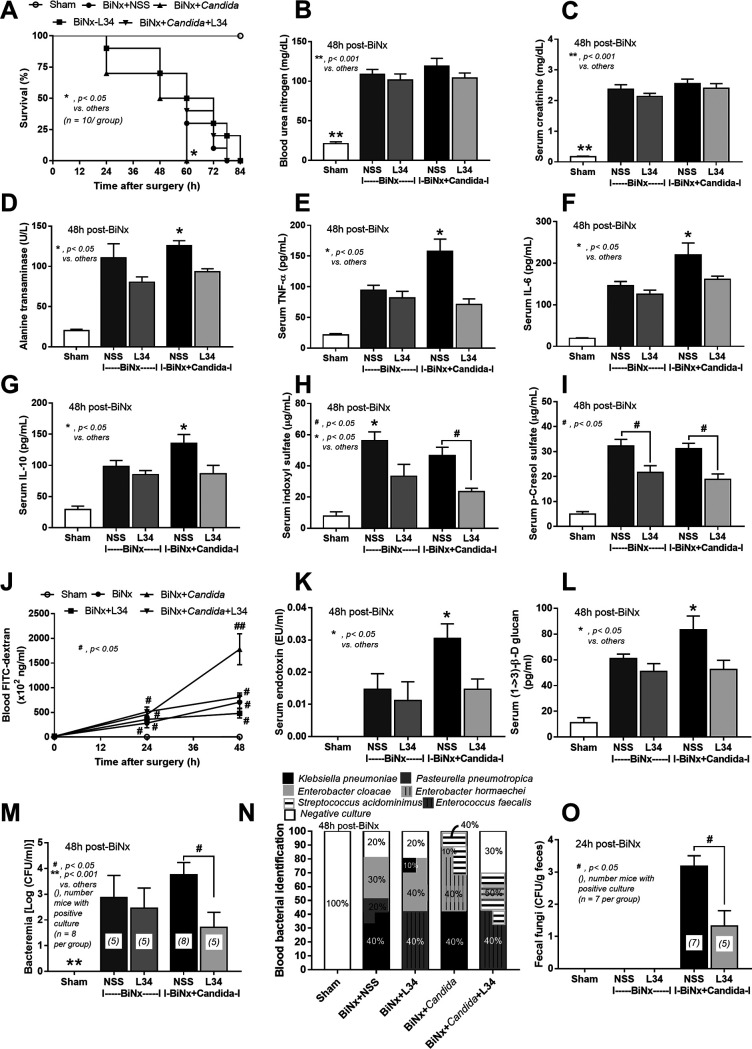
Characteristics of sham mice or bilateral nephrectomy (BiNx) mice with normal saline (NSS) or *Candida* administration with or without Lactobacillus rhamnosus L34 (L34) administration as determined by survival analysis (A); kidney injury by blood urea nitrogen and serum creatinine (B and C); alanine transaminase (D); serum cytokines (F and G); gut-derived uremic toxin by indoxyl sulfate and *p*-cresol (H and I); gut leakage by FITC-dextran, endotoxemia, serum (1→3)-β-d-glucan, and bacteremia (J to M); identification of blood bacteria (O) (48 h after BiNx for panels A to O); and fecal fungal burdens (24 h after BiNx) (P) (*n* = 6 to 8/group).

All BiNx mice, with or without *Candida*, were in the moribund stage within 60 h or 78 h, respectively, supporting enhanced BiNx severity by gut fungi (Fig. 10A). In parallel, *Candida* administration in BiNx enhanced liver injury (serum alanine transaminase), systemic inflammation (serum cytokines), and the severity of gut leakage (FITC-dextran, endotoxemia, and glucanemia) but not renal function (blood urea nitrogen [BUN] and serum creatinine [SCr]), gut-derived uremic toxins (indoxyl sulfate and *p*-cresol), and bacteremia in comparison with BiNx without *Candida* (Fig. 10B to M). Due to the influence of gut bacteria on the production of indoxyl sulfate and *p*-cresol (37), the higher levels of both gut-derived toxins in BiNx over those in sham mice (Fig. 10H and I) implied alterations of the gut microbiota from uremia. In addition, 20% of blood cultures were negative in mice with BiNx alone, while all BiNx-*Candida* mice demonstrated bacteremia (Fig. 10N), implying the influence of fungus-induced alterations in the gut microbiota. The predominant bacteria in the blood of BiNx-*Candida* mice were Klebsiella pneumoniae, Enterobacter hormaechei, and Streptococcus acidominimus, while the predominant bacteria in the blood of BiNx-alone mice were K. pneumoniae, Pasteurella pneumotropica, and Enterobacter cloacae (Fig. 10N).

**FIG 11:**
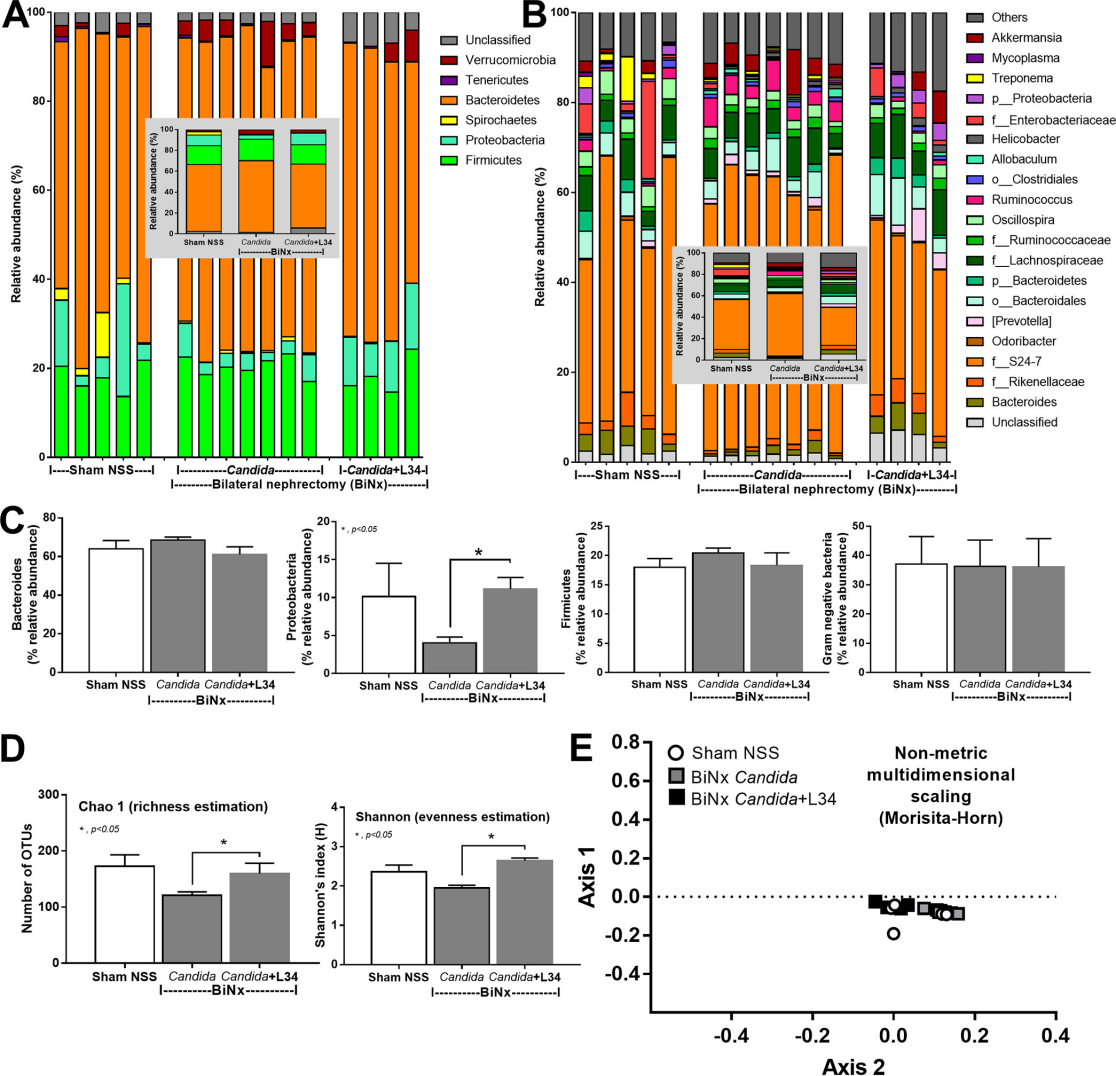
Fecal microbiota analysis of sham mice or bilateral nephrectomy (BiNx) mice with normal saline (NSS) or *Candida* administration with or without Lactobacillus rhamnosus L34 (L34) administration at 48 h postoperation by the relative abundance of bacteria at the phylum and genus levels (A and B) (inset graphs show average values) with graph presentations of bacterial phyla and total fecal Gram-negative bacteria evaluated by bacterial phylum (C); alpha diversity presented by Chao 1, Shannon index, and total operational taxonomic units (OTUs) (D); and beta diversity (nonmetric multidimensional scaling based on Morisita-Horn dissimilarity indices) (E).

Nevertheless, Lactobacillus rhamnosus L34 (L34) attenuated the severity of BiNx-*Candida*, as moribund mice presented as late as 78 h versus 60 h after BiNx in BiNx-*Candida* L34 versus BiNx-*Candida*, respectively (Fig. 10A). In BiNx-*Candida*, L34 attenuated liver injury, systemic inflammatory responses, gut-derived uremic toxins, gut leakage, bacteremia, and fecal fungi in comparison with BiNx-*Candida* without L34 (Fig. 10). However, L34 did not attenuate the severity of BiNx without *Candida* administration (Fig. 10). In fecal microbiome analyses, L34 increased only *Proteobacteria* in BiNx-*Candida* mice to a level similar to that in sham mice without the alteration of the total Gram-negative bacterial abundance in feces (Fig. 11A to C). However, L34 normalized the gut microbiota in BiNx mice, as demonstrated by the restoration of bacterial variety in feces (bacterial richness and evenness estimation) (Fig. 11D). There was a close proximity of taxonomic abundance profiles by β-diversity analysis (demonstrating similar types of bacteria) among sham and BiNx with or without *Candida* (Fig. 11E).

## DISCUSSION

Candida albicans in mouse feces is detectable only by PCR (38) but not by culture (17), which differs from human conditions (18). Thus, C. albicans administration in bilateral nephrectomy (BiNx) mice is necessary to induce fungal presentation in feces. Indeed, *Candida* administration in BiNx mice worsened uremia through enhanced systemic inflammation by LPS and (1→3)-β-d-glucan (BG) from gut translocation. Our data support the clinical importance of gut fungi in acute uremia.

### Uremia enhanced fungal and bacterial growth, possibly through impaired mucosal immunity and intestinal inflammation.

Because of the inadequate excretion of uremic toxins after renal dysfunction, several uremic toxins (such as urea, uric acid, and phosphorus) are transferred into the intestine, an alternative organ for toxin excretion, causing an alteration in the intestinal environment that is favorable for some groups of bacteria. Indeed, the impacts of some uremic toxins are as follows: (i) basification of the intestinal environment occurs due to the hydrolysis of urea by urease-producing bacteria (39–41), (ii) some uremic toxins (uric acid and urea) are alternative substrates for some microbes (42), (iii) a reduction of short-chain-fatty-acid bacteria from uremic toxins induces intestinal permeability defects (41, 43), and (iv) a direct effect of uremia on enterocytes induces cell edema, inflammation, and loss of mucus that affect some groups of bacteria (44). However, data on the influence of uremic toxins on gut fungi are still lacking despite the increased prevalence of systemic fungal infection in patients with uremia (45). Here, uremia enhanced fungal growth in BiNx mice 24 h after oral administration. This is perhaps due to uremia-induced gut inflammation that reduced mucosal immunity (46, 47), similar to other models (13, 30, 48, 49). Indeed, uremia-induced inflammation in the gut was demonstrated by increased intestinal cytokines 48 h after BiNx, which was more prominent with BiNx-*Candida* than with BiNx alone. Fungal gavage in healthy mice (*Candida*-sham) did not induce gut inflammation, highlighting the role of mucosal defense mechanisms. However, uremia-induced fungal overgrowth was not potent enough to spontaneously induce positive fungal cultures in mouse feces without *Candida* administration. Fungal gavage was necessary to induce fungal presentation in the gut that was high enough for a positive fungal culture.

In addition, data on the alterations in the gut microbiota from acute uremia are limited despite the well-known alterations by chronic uremia (50). Indeed, acute uremia in BiNx mice mildly affected the fecal microbiome in comparison with sham mice. Meanwhile, BiNx-*Candida* in comparison with BiNx alone increased *Bacteroides* species, Gram-negative anaerobes with a capacity for colitis induction (51); reduced *Firmicutes*, Gram-positive anaerobes with potential health benefits (52); and decreased bacterial diversity, an indicator of gut unhealthiness (52). Because fecal Gram-negative bacterial burdens between groups were similar, higher endotoxemia in BiNx-*Candida* mice than in BiNx-alone mice should be responsible for *Candida-*enhanced gut permeability defects but not the increased LPS in gut contents. Despite the major alterations in the gut microbiota in patients with acute uremia as previously reported (53), the alterations in the gut microbiota in BiNx mice were subtle but enhanced by *Candida* administration. The discrepancy between the data from patients (53) and those from mice (here) also suggests a possible effect of gut *Candida* on uremia.

### *Candida* presentation in the gut enhances systemic inflammation in bilateral nephrectomy mice through gut leakage.

*Candida* shortened the life span of BiNx mice, as all mice in the BiNx-*Candida* group reached the moribund stage faster than mice with BiNx alone. This was possibly due to enhanced systemic inflammation (serum cytokines) and liver injury but not uremia (similar serum creatinine levels). *Candida* worsened the intestinal permeability defect of BiNx mice; allowed the gut translocation of organismal molecules into blood circulation through the portal vein and the lymphatic system into the liver and mesenteric lymph nodes, respectively; and activated inflammation through hepatocytes and macrophages (54, 55). Interestingly, uremia-induced gut leakage in BiNx mice was severe enough for the translocation of viable bacteria to several internal organs, possibly due to uremia-induced enterocyte apoptosis. Indeed, enterocyte cell death causes a larger defect on the intestinal mucosa than tight junction injury (6). Among all organs with bacterial presentation, detectable bacteria in mesenteric lymph nodes supported gut bacterial translocation (6) in acute uremia of BiNx, but gut leakage was not severe enough for the translocation of viable fungi.

Furthermore, viable bacteria, LPS, and BG from gut translocation in BiNx-*Candida* mice enhanced serum cytokine production (16), which was aggravated by the absence of urinary cytokine excretion in BiNx (56). The accumulated cytokines in serum induced higher levels of cytokine production from hepatocytes and macrophages (57), in a vicious cycle, and induced more severe liver injury (liver enzymes and histology) at 60 h postsurgery in BiNx-*Candida* mice than in BiNx-alone mice.

### Additive effect of endotoxin (LPS) and (1→3)-β-d-glucan on hepatocytes and macrophages.

BiNx-*Candida* mice demonstrated higher levels of LPS and BG in serum than did BiNx-alone mice. Interestingly, LPS and BG synergistically induced inflammation through TLR-4 and Dectin-1, respectively (16), which are expressed on both hepatocytes and macrophages (58, 59). As such, the additive inflammatory effects of LPS plus BG (LPS+BG), compared with each separate molecule, on hepatocytes (HepG2) and macrophages (RAW264.7) were demonstrated by enhanced cytokine production, especially TNF-α and IL-10. The inflammatory amplification of BG on the LPS response was demonstrated by the prominent versus low cytokine levels with LPS+BG versus BG alone, respectively. In addition, uremic toxins amplified the effect of LPS+BG as incubation with serum from mice 48 h after BiNx (uremia serum) increased cytokine levels compared with the use of sham control serum (CtlS), supporting uremia-induced inflammation (2). Since (i) the liver is directly activated by organismal molecules from gut leakage and (ii) data on hepatocyte energy metabolism are still limited, extra flux analysis was explored. Accordingly, both glycolysis and mitochondrial function of hepatocytes were not altered by activation with LPS or BG alone, while LPS+BG further reduced the functions that might be associated with hepatocyte injury (60, 61). Interestingly, only LPS+BG, but not each molecule alone, altered hepatic energy metabolism, implying a synergistic effect of these molecules.

On the other hand, the enhancement of proinflammatory macrophage glycolysis by LPS (62) and LPS+BG synergy on macrophages have been mentioned previously (63–66), while LPS+BG did not significantly enhance the characteristics of M1 proinflammatory macrophages as determined by the similar expression levels of *iNOS* and *IL-1*β between LPS+BG and LPS alone. Uremic toxins from BiNx serum amplified the expression of *iNOS*, *IL-1*β, and *TNF*-α, supporting uremia-induced proinflammatory macrophages (2). Hence, our data supported the impact of fungi and Gram-negative bacteria on the gut as (i) an endogenous source of organismal molecules, (ii) an inducer of gut leakage, and (iii) a synergy on the proinflammatory responses against bacterial plus fungal molecules.

### Probiotic treatment in acute uremia, attenuation of gut leakage, and microbiota alterations in the gut.

Although the benefit of probiotics in chronic kidney disease, especially *Lactobacillus* spp., has been demonstrated (67), the effect on acute uremia from bilateral nephrectomy with or without *Candida* administration has never been explored. *Lactobacillus* species attenuates uremia (67), and the benefits of L. rhamnosus L34 (L34) against gut microbiota alterations and gut leakage through anti-inflammatory products and the reduction of fecal pathogenic bacteria have been demonstrated (15, 27). Indeed, L34 attenuated the severity of BiNx*-Candida* but not BiNx alone, as determined by survival analysis, liver injury, serum cytokines, and gut-derived uremic toxins, possibly by the reduced gut fungi and the decreased severity of gut leakage. The normalization of the gut microbiota in BiNx by L34 was illustrated by an improvement in bacterial diversity, including bacterial richness (higher number of bacterial sequences) and bacterial evenness (increased variety of bacterial species). However, L34 did not alter fecal total Gram-negative bacteria, a source of fecal LPS, and the ratio of *Bacteroides*/*Firmicutes* in feces, an indicator of microbiota alterations (68), but increased *Proteobacteria*. Perhaps, L34-induced *Proteobacteria* might be responsible for the management of nitrogen waste products from uremia by the nitrogen fixation properties of bacteria in this group (69). Indeed, gut-derived uremic toxins were decreased by L34 administration. Of note, K. pneumoniae, a pathogenic member of the *Proteobacteria*, and Streptococcus acidominimus, a viridans group streptococcus with possible pathogenicity (70), in blood were demonstrated only in BiNx mice without L34 administration, indicating a reduction in pathogenic bacteremia. Hence, L34 normalized the gut microbiota in the components of both bacteria and fungi, attenuated gut leakage, and improved BiNx conditions. In translation, the manipulation of gut leakage and/or fungal burdens by probiotics is an interesting strategy as an adjuvant therapy for acute uremia.

In conclusion, *Candida* administration enhanced the severity of BiNx in mice through microbiota alterations, gut leakage, and gut translocation of organismal molecules, especially LPS and BG. The additive inflammatory effects of BG and LPS together with amplification by uremic toxins against hepatocytes and macrophages enhanced the severity of BiNx-*Candida* mice, which was attenuated by a probiotic. Further studies of L34 in patients with acute uremia are of interest.

## MATERIALS AND METHODS

### Animals.

The animal care and use protocol according to the U.S. National Institutes of Health (NIH) was approved by the Institutional Animal Care and Use Committee of the Faculty of Medicine, Chulalongkorn University, Bangkok, Thailand. Male 8-week-old C57BL/6 mice from Nomura Siam International (Pathumwan, Bangkok, Thailand) were purchased.

### *Candida* administration in a bilateral nephrectomy model.

To explore the impact of gut fungi in acute uremia, Candida albicans was administered to bilateral nephrectomy (BiNx) mice. C. albicans from the American Type Culture Collection (ATCC 90028) (Fisher Scientific, Waltham, MA, USA) was cultured overnight on Sabouraud dextrose broth (SDB) (Oxoid, Hampshire, UK) at 35°C for 48 h before enumeration using a hemocytometer, and C. albicans at 1 × 10^6^ CFU in a 0.5-ml phosphate buffer solution (PBS) or PBS alone was orally administered every morning (8 a.m.) for 7 days to induce *Candida* presentation in the gut. Next, BiNx was performed 6 h (2 p.m.) after the last dose of fungi by abdominal incision according to previous publications (57, 71, 72). Briefly, renal capsules were separated before kidney removal with ligation of renal vessels and ureters. In the sham group, renal vessels and ureters were identified only by abdominal incision before closing the abdomen. Fentanyl at 0.03 mg/kg of body weight in 0.5 ml of a normal saline solution (NSS) was subcutaneously administered after the operation for analgesia and fluid replacement. In addition, Lactobacillus rhamnosus L34 (L34) was isolated from Thai infant feces (25) and cultured on de Man-Rogosa-Sharpe (MRS) agar (Oxoid, Hampshire, UK) under anaerobic conditions with gas generation sachets (AnaeroPack-Anaero; Mitsubishi Gas Chemical Co., Inc., Japan) at 37°C for 48 h before quantitative preparation by the determination of the optical density at 600 nm (OD_600_). Additionally, L34 at 1 × 10^8^ CFU in 0.5 ml PBS or PBS alone was administered daily at 12 p.m. for 7 days before the BiNx operation 2 h after the last dose of L34. Mice were sacrificed under isoflurane anesthesia, with blood and organ collection. Organs were (i) processed for bacterial and fungal culture to determine the translocation of organisms from intestines to the internal organs, (ii) snap-frozen in liquid nitrogen and kept at −80°C before tissue cytokine determination, (iii) kept in 10% formalin for histological processes, and (iv) put in tissue frozen in optimal cutting temperature (OCT) compound (Tissue-Tek OCT compound; Sakura Finetek USA, Inc., Torrance, CA, USA) for fluorescence microscopic evaluation. Feces from all parts of the colon were combined and collected for fecal microbiome analysis and fecal fungal burden determinations. For survival analysis, mice were further observed, and moribund mice were sacrificed. Of note, the data at 0 h were collected 3 days before the operation and were used as the baseline values.

### Mouse blood sample analysis, gut leakage measurement, and fecal fungal burden.

Renal injury, blood urea nitrogen (BUN), and serum creatinine (SCr) were determined by QuantiChrom urea (catalog no. DIUR-500) and creatinine (catalog no. DICT-500) assays (Bioassay, Hayward, CA, USA). Liver damage and serum cytokines were evaluated by using an EnzyChrom alanine transaminase assay (catalog no. EALT-100; Bioassay) and an enzyme-linked immunosorbent assay (ELISA) for mouse (Invitrogen, Carlsbad, CA, USA), respectively. Gut-derived uremia toxins, indoxyl sulfate, and *p*-cresol sulfate were measured by high-performance liquid chromatography (HPLC) (Alliance 2695; Waters, Zellik, Belgium). Gut leakage was determined by (i) detection of fluorescein isothiocyanate-dextran (FITC-dextran), a nonabsorbable high-molecular-weight molecule in serum, after oral administration; (ii) spontaneous serum elevation of (1→3)-β-d-glucan (BG) and endotoxin (LPS), major cell wall components of fungi and Gram-negative bacteria, respectively; and (iii) detection of viable organisms (bacteria and fungi) in mesenteric lymph nodes and internal organs as previously described (14, 15, 30, 73). Briefly, for FITC-dextran assays, 0.5 ml of FITC-dextran (molecular weight, 4.4 kDa) (Sigma-Aldrich) at 25 mg/ml was orally administered 3 h prior to sacrifice, and blood collected at sacrifice was analyzed by fluorescence spectroscopy (Varioskan Flash; Thermo Scientific) at excitation and emission wavelengths of 485 and 528 nm, respectively, with a standard curve of FITC-dextran. Serum BG and LPS levels were determined by using Fungitell (Associates of Cape Cod, Inc., East Falmouth, MA, USA) and HEK-Blue LPS detection (InvivoGen, San Diego, CA, USA), respectively. When the values of BG and LPS were <7.8 and <0.01 endotoxin units (EU)/ml, respectively, they were recorded as 0 due to being beyond the lower limit of the standard curve. For the organism burdens, the homogenized organs in PBS or 25 μl of blood was directly spread onto blood agar or 0.1% chloramphenicol in Sabouraud dextrose agar (SDA) (Oxoid, Germany) for the determination of bacteria and fungi, respectively, and incubated at 37°C for 24 h and at 35°C for 72 h, respectively, before colony enumeration. The bacterial colonies were identified by mass spectrometry analysis (Vitek MS; bioMérieux SA, Marcy-l’Etoile, France) according to routine hospital protocols. In addition, fecal samples were suspended with PBS at a ratio of 100 μg per 1 μl, serially diluted before plating onto SDA (Oxoid), and incubated at 35°C for 72 h before colony enumeration of fungi.

### Histological analysis and cytokines of the internal organs.

Liver tissue was fixed in 10% formalin, embedded in paraffin, cut to a 4-μm thickness, and stained with periodic acid-Schiff (PAS) stain and hematoxylin-eosin (H&E). Hepatic injury was defined as congestion, cellular degenerative changes, cytoplasmic vacuolization, leukocyte infiltration, or cellular necrosis. The degree of injury was estimated at a ×200 magnification using 10 randomly selected fields for each animal using the following scores per examination field: 0 for an area of damage of <10%, 1 for an area of damage of 10 to 25%, 2 for damage involving 25 to 50% of the area, 3 for damage involving 50 to 75% of the area, and 4 for 75 to 100% of the area being affected (modified from a method reported in previous publication [57]). In addition, localized inflammation in the liver and intestines was determined. The ileum and ascending colon were collected 2 cm proximally and distally to the cecum, respectively. Next, the tissues were weighed, sonicated thoroughly, put into 1 ml PBS per g tissue, and centrifuged, and the supernatant was collected for cytokine measurement by an ELISA (Invitrogen).

Because of the prominent inflammation in the intestines of previously reported *Candida*-administered mouse models (14, 15), the tight junctions from the ileum and colon were determined to be representative of the small and large bowels, respectively. The intestines were put into tissue frozen in OCT compound (Tissue-Tek OCT compound; Sukura Finetek, Inc., CA, USA) prepared in 5-μm-thick frozen tissue sections. The slides were fixed with acetone for 10 min and blocked with a solution containing 1% bovine serum albumin (BSA) and 10% fetal bovine serum (FBS) in PBS. Blocked slides were stained with primary antibody against enterocyte tight junction molecules of zonula occludens 1 (ZO-1) (catalog no. 61-7300; Thermo Fisher Scientific, IL, USA) (1:200). The slides were washed three times with PBS and then incubated with the secondary antibody Alexa Fluor 546 goat anti-rabbit IgG (catalog no. A-11035; Life Technologies, USA) (1:200) at 23°C for 1 h. Slides were washed as described above and then stained with 4′,6-diamidino-2-phenylindole (DAPI; BioLegend, USA) (1:1,000) for 15 min. After mounting (Prolong; Life Technologies), slides were visualized with a Zeiss LSM 800 confocal microscope (Carl Zeiss, USA). Furthermore, the intestinal tight junction and apoptotic cells were also determined by immunohistochemistry modified from a previously reported protocol (7). Briefly, 10% formalin-fixed and paraffin-embedded sections of the ileum and colon were stained with anti-ZO-1 (catalog no. 61-7300; Thermo Fisher Scientific) (1:200) or anti-cleaved caspase-3 (Asp175) (catalog no. 9661; Cell Signaling Technology, Beverly, MA) (1:400) at 23°C for 1 h. Sections were then incubated at 23°C with secondary biotinylated goat anti-rabbit antibody (Cell Signaling Technology) (1:200) for 30 min before visualization. The semiquantitative analysis of the intensity from 10 random fields per slide was performed separately by 2 pathologists. As such, the expression of ZO-1 protein at the intestinal villi was evaluated at a ×200 magnification as previously reported (74), according to the following scale: 0 for an absence of labeling, 1 for mild labeling, and 2 for robust labeling. In parallel, the average number of enterocytes with apoptosis counted at a ×200 magnification was evaluated and expressed as the number of positive cells per high-power field.

### Fecal microbiome analysis.

Fecal microbiota analysis was performed according to methods reported in previous publications (15, 30). Briefly, total DNA from feces of individual mice (0.25 g) was extracted by using a power DNA isolation kit (MoBio, Carlsbad, CA, USA), and metagenomic DNA quality was determined by agarose gel electrophoresis and nanodrop spectrophotometry. Universal prokaryotic forward primer 515F (5′-GTGCCAGCMGCCGCGGTAA-3′) and reverse primer 806R (5′-GGACTACHVGGGTWTCTAAT-3′), with appended 50 Illumina adapter and 30 Golay barcode sequences, were used for 16S rRNA gene V4 library construction. Triplicate PCRs were performed (25-μl mixtures), and each reaction mixture consisted of 1× EmeraldAmp1 GT PCR master mix (TaKaRa), 0.2 μM each primer, and the metagenomic DNA (75 ng). A GenepHlow gel extraction kit (Geneaid Biotech Ltd., New Taipei City, Taiwan) was used for purifying 16S rRNA from an agarose gel, and quantification was performed using PicoGreen (Invitrogen, Eugene, OR, USA). Each sample (240 ng) was applied to the MiSeq300 sequencing platform (Illumina, San Diego, CA, USA) with previously described index sequences (75). Mothur’s standard quality screening operating procedures for the MiSeq platform with aligned and assigned taxa (operational taxonomic units [OTUs]) based on default parameters were used (76).

### Hepatocyte and macrophage cell line experiments.

HepG2 human hepatoma cells (ATCC HB-8065) and RAW264.7 mouse macrophage cells (ATCC TIB-71) from the American Type Culture Collection (Manassas, VA, USA) were maintained in Dulbecco’s modified Eagle medium (DMEM) supplemented with 10% heat-inactivated FBS (Gibco, Life Technologies Ltd., Paisley, UK), 1% sodium pyruvate, 1% nonessential amino acids (NEAA) (minimal essential medium [MEM] NEAA; Gibco, USA), and 1% penicillin/streptomycin antibiotic (Pen Strep; Gibco, Life Technologies Corporation, Grand Island, NY, USA) and incubated at 37°C in a humidified 5% CO_2_ incubator for 24 h. Next, HepG2 cells at 2 × 10^5^ cells/ml in 96-well flat-bottomed tissue culture plates were incubated with purified lipopolysaccharide (LPS) (1 μg/ml) from Escherichia coli O26:B6 (Sigma-Aldrich, St. Louis, MO, USA) alone or in combination with CM-Pachyman (100 μg/ml) (Megazyme, Bray, Ireland), as a representative of BG, and coincubated with or without heat-treated mouse serum (100 μg/ml) from the sham group (control serum [CtlS]) or the BiNx group (BiNxS) for 72 h. Mouse serum 48 h after sham (CtlS) or BiNx (BiNxS) treatment was heated at 56°C for 30 min, and 100 μl/well was used for incubation. The total volume in each well was 200 μl. In parallel, a similar protocol was used in RAW264.7 cells except for (i) a smaller number of cells in each well (1 × 10^5^ cells/well), (ii) a shorter incubation (for 6 h), and (iii) a lower LPS dose (100 ng/ml), due to the toxicity of high-dose LPS toward macrophages (77). After that, supernatant cytokines were determined using an ELISA for human cytokines (R&D Systems, Minneapolis, MN, USA) (TNF-α, IL-8, and IL-10) in HepG2 cell experiments and by an ELISA for mouse cytokines (Invitrogen) (TNF-α, IL-6, and IL-10) in RAW264.7 cell experiments. Notably, supernatant IL-8 was measured in HepG2 cells due to the ineffectiveness of HepG2 cells in IL-6 production (78).

In addition, to further explore the impact of LPS plus BG (LPS+BG) on hepatocytes, the energy metabolism profiles with glycolysis estimation by the extracellular acidification rate (ECAR) and mitochondrial oxidative phosphorylation by the oxygen consumption rate (OCR) were determined using Seahorse XFp analyzers (Agilent, Santa Clara, CA, USA) with Seahorse Wave 2.6 software (79). Energy parameters were calculated by the generator program report based on the following equations: respiratory capacity (maximal respiration) = OCR between carbonyl cyanide-4-(trifluoromethoxy)-phenylhydrazone (FCCP) and antimycin A/rotenone − OCR after antimycin A/rotenone, respiratory reserve = OCR between FCCP and antimycin A/rotenone − OCR before oligomycin, glycolysis = ECAR between glucose and oligomycin − ECAR before glucose administration, and glycolysis capacity = ECAR between oligomycin and 2-deoxy-d-glucose (2-DG) − ECAR before glucose administration.

Since macrophage polarization is associated with pro- or anti-inflammation as M1 or M2 polarization, respectively, the expression of macrophage genes was determined by PCR. As such, total RNA was prepared by using an RNeasy minikit (Qiagen, Hilden, Germany) and a high-capacity reverse transcription assay (Applied Biosystems, Warrington, UK) on an Applied Biosystems 7500 Real-165 Time PCR system (Applied Biosystems) using SYBR green PCR master mix (Applied Biosystems). Relative quantitation normalized to β-actin (an endogenous housekeeping gene) by the comparative threshold cycle method (2^−ΔΔ^*^CT^*) was performed. The list of primers for PCR is presented in Table 1.

**TABLE 1:** List of primers^*a*^

Target	Primer sequence	
	Forward	Reverse
Arg-1	5′-CTTGGCTTGCTTCGGAACTC-3′	5′-GGAGAAGGCGTTTGCTTAGTTC-3′
iNOS	5′-ACCCACATCTGGCAGAATGAG-3′	5′-AGCCATGACCTTTCGCATTAG-3′
IL-1β	5′-GAAATGCCACCTTTTGACAGTG-3′	5′-TGGATGCTCTCATCAGGACAG-3′
TNF-α	5′-CCTCACACTCAGATCATCTTCTC-3′	5′-AGATCCATGCCGTTGGCCAG-3′
Arg-1	5′-CTTGGCTTGCTTCGGAACTC-3′	5′-GGAGAAGGCGTTTGCTTAGTTC-3′
FIZZ-1	5′-GCCAGGTCCTGGAACCTTTC-3′	5′-GGAGCAGGGAGATGCAGATGAG-3′
TGF-β	5′-CAGAGCTGCGCTTGCAGAG-3′	5′-GTCAGCAGCCGGTTACCAAG-3′
β-Actin	5′-CGGTTCCGATGCCCTGAGGCTCTT-3′	5′-CGTCACACTTCATGATGGAATTGA-3′

a Arg-1, arginase 1; iNOS, inducible nitric oxide synthase; IL-1β, interleukin-1β; TNF-α, tumor necrosis factor alpha; FIZZ-1, Resistin-like molecule α; TGF-β, transforming growth factor β.

### Statistical analysis.

Means ± standard errors (SE) were used for data presentation, and the differences between groups were examined for statistical significance by one-way analysis of variance (ANOVA) followed by Tukey’s analysis or Student’s *t* test for comparisons of multiple groups or 2 groups, respectively. Survival analysis was performed by a log rank test. All statistical analyses were performed with SPSS 11.5 software (SPSS, Chicago, IL, USA) and GraphPad Prism version 7.0 software (GraphPad, La Jolla, CA, USA). A *P* value of <0.05 was considered statistically significant. Repeated-measures ANOVA was used for the analysis of time course data.
